# A Promising Use of Trimethyl Chitosan for Removing *Microcystis aeruginosa* in Water Treatment Processes

**DOI:** 10.3390/microorganisms10102052

**Published:** 2022-10-18

**Authors:** Leda Giannuzzi, Julián Bacciadone, Graciela L. Salerno

**Affiliations:** 1 Centro de Investigación y Desarrollo en Criotecnología de Alimentos (CIDCA-CONICET), La Plata 1900, Argentina; 2 Fundación Para Investigaciones Biológicas Aplicadas (FIBA), Vieytes 3103, Mar del Plata 7600, Argentina

**Keywords:** cyanobacterial blooms, *Microcystis aeruginosa*, chitosan, trimethylchitosan, a second order quadratic model

## Abstract

The increase in cyanobacterial blooms linked to climate change and the eutrophication of water bodies is a global concern. The harmful cyanobacterium *Microcystis aeruginosa* is one of the most common bloom-forming species whose removal from fresh water and, in particular, from that used for water treatment processes, remains a crucial goal. Different biodegradable and environmentally friendly coagulants/flocculants have been assayed, with chitosan showing a very good performance. However, chitosan in its original form is of limited applicability since it is only soluble in acid solution. The objective of this work was therefore to test the coagulant/flocculant capacity of trimethylchitosan (TMC), a chitosan derivative produced from residues of the fishing industry. TMC has a constitutively net positive charge enabling it to remain in solution regardless of the pH. Results show that even at alkaline pHs, common during cyanobacterial blooms, TMC is effective in removing buoyant cyanobacteria from the water column, both in test tube and Jar-Test experiments. Cell integrity was confirmed by fluorescent stain and electron microscopy. Our findings lead us to conclude that the use of TMC to remove bloom cells early in the treatment of drinking water is both feasible and promising.

## 1. Introduction

Toxic cyanobacterial blooms in freshwater systems are currently increasing globally in frequency, distribution, magnitude, and duration, due to anthropogenic eutrophication and climate change [1,2,3,4]. The impact of these events depends mainly on the extent and nature of the bloom and the presence of cyanotoxins such as microcystins (MCs), saxitoxins, and cylindrospermopsin. When blooms affect water supply systems, they become a major concern for human and animal health [4,5]. Another important problem caused by cyanobacterial blooms is that their cells interfere with drinking water production, causing filter-clogging, risks posed by disinfection byproducts, high residual levels of coagulants, unpleasant taste and odor, toxin generation, and a high organic content in raw material [6,7,8]. The elimination of cells is therefore a crucial step in the production of drinking water; cell removal not only facilitates the process but also can also significantly reduce the concentration of unpleasant tastes and odors, and the presence of toxic intracellular metabolites.

Among the bloom-forming cyanobacteria, *Microcystis aeruginosa* is the most common and widespread species found in freshwater bodies around the world and produces stable toxins as secondary metabolites [9]. MCs have a harmful impact on both ecological systems and humans. They specifically inhibit protein phosphatases, leading to increased phosphorylation in human cells, the liver being a target organ for their action. However, besides being hepatotoxins, MCs are also classified by the International Agency for Research on Cancer (IARC) as possibly carcinogenic to humans (group 2B). MCs usually show persistence, are stable in intact *Microcystis* cells, and are shown to degrade upon release [8]. However, in ageing blooms or after chemical treatments, MC concentrations may increase. The elimination of *M. aeruginosa* cells from water entering treatment plants maintaining cell integrity is therefore crucial [10].

The risk of cyanobacterial toxins in drinking water treatment plants (DWTP) can be minimized using a multi-barrier approach involving treatment of the water feeding the plant, reducing the level of toxin release from cells, optimizing the toxin elimination process, and monitoring the water at the outlet of the plant [10]. A key step at the beginning of the process is the removal of cells from the water column, which is usually carried out by chemical coagulation/flocculation (C/F), terms usually used interchangeably [11]. In the C/F procedure, coagulation occurs first, generating small colloids suspended in the water that become destabilized, followed by flocculation in which the particles aggregate to form flocs, which can be removed by sedimentation. The C/F process is now the most used process in water treatment since it has been shown to be safe and economical compared to other algae removal methods, and generally prevents cell damage by the subsequent release of toxins, a major concern for DWTP operators and water supply managers. Several types of C/F materials have been explored, such as inorganic salts of multivalent metals (e.g., polyaluminum chloride, ferric chloride, aluminum sulfate, and polymerized ferrous sulfate), and synthetic and natural organic polymers (e.g., polyacrylamide derivatives and chitosan, respectively). Chemical treatments have been widely used to control cyanobacterial cells in view of their low cost and high efficiency [12]. However, various chemicals such as copper sulfate and potassium permanganate cause cell lysis and the subsequent release of toxins [13]. In addition, inorganic coagulants must be used in high dosages for effective removal and generate secondary pollution, since the resulting sludge contains heavy metals, toxic to the environment and humans [14]. On the other hand, the use of polyacrylamide for C/F is the main source of drinking-water contamination by acrylamide monomer, which is considered a potential human carcinogen and mutagen, with low biodegradability [15,16]. Consequently, the development and application of natural polymers, such as chitosan, has been promoted as a possible alternative to traditional coagulants [17,18]. Chitosan is an advantageous agent for any water treatment, especially for drinking water, as it is considered non-toxic and safe [11].

Chitosan is a linear copolymer of 2-acetamido-2-deoxy-D-glucose (N-acetyl glucosamine, GlcNAc), and 2-amino-2-deoxy-D-glucose with ß-D-(1→4) glycoside linkages. It can be extracted from shellfish and crustaceans by enzymatic or chemical deacetylation of chitin, the most abundant aminopolysaccharide polymer occurring in nature [19]. The degree of N-acetylation has been used to differentiate chitin from chitosan [20]. 

The main natural sources of chitin are shrimp and crab shells, abundant by-products of the food-processing industry. The different hydrolysis conditions of chitin give rise to chitosan with different chain lengths, degrees of deacetylation, and molecular weight. Those biopolymers are used in different applications in biomedicine, food processing, and wastewater treatment [11]. In water treatment in particular, chitosan has been extensively applied as a non-toxic and biodegradable compound, acting simultaneously as a coagulant and flocculant, since under appropriate pH conditions its amino groups are protonated, generating a positive charge that neutralizes the negative charge of the cell membrane [11,21,22]. The use of chitosan for the C/F of *M. aeruginosa* was shown to effectively remove 99% of the cells while maintaining their integrity [11,18,23,24].

However, a significant drawback to the application of chitosan in its original form in DWTP is that it is soluble only in an acid solution due to its strong intermolecular and intramolecular hydrogen bonds [25]. 

A great disadvantage of the application of chitosan for cell coagulation in water treatment is that its solubilization requires an acidic pH [25]. The modification of chitosan to improve its solubility has been a focus of research in recent years. Jin et al. [26] proposed the use of chitosan quaternary ammonium salt, a water-soluble chitosan derivative, for the removal of *M. aeruginosa* cells from drinking water. 

Chitosan derivate (CTS) was used with montmorillonite to form nanocomposites (CTS/NMMT) for the removal cells of *M. aeruginosa* [27]. The Box–Behnken response surface model was used to study various factors such as the weight ratio of NMMT to CTS and the necessary agitation time for the efficient removal of *M. aeruginosa*. The highest removal was found to be 94.9% using the nanocomposite. 

The effect of different doses of a composite coagulant CTSAC (Chitosan and Aluminum Clorine premixed) on removal of *M. aeruginosa* cells was studied by Ma et al., 2016 [28]. Using the response surface methodology (RSM), the coagulation processes were modeled and optimized. The investigated coagulant produced a 97% reduction in cells and achieved better removal effects than coagulant applied individually. 

A novel buoyant-bead flotation method using chitosan-coated fly ash cenospheres was developed to remove HABs in freshwater. An optimized removal efficiency of 98.50% for *M. aeruginosa* was reached at a pH of 6.0 [29].

Another study found that low concentrations of chitosan (2 mg L^−1^) combined with a ballast may be sufficient to flock and sink cyanobacteria (*M. aeruginosa*) effectively in freshwaters [30].

In Argentina, *M. aeruginosa* blooms are a growing health and environmental concern [31]. Our research aims at providing economical, safe, and ecological solutions for water treatment. Given that crustacean fishing is an important marine resource in the southwest Atlantic waters of Argentina, the processing residues, containing chitosan, constitute a very good source of raw material. In the present study, we evaluate the capacity of trimethylated chitosan (TMC), synthesized from a chitosan obtained from by-products of the sea food industry, to remove *M. aeruginosa* cells in a wide pH range for use in drinking water purification processes.

## 2. Materials and Methods

### 2.1. Biological Material and Culture

Two *M. aeruginosa* strains were used in this study: PCC 7806, from the Pasteur Culture Collection (France), and CAAT 2005-3 (FIBA collection, Mar del Plata, Argentina), a native strain isolated from Buenos Aires Province, Argentina [32]. *M. aeruginosa* cells were cultured in BG-11 medium [33] supplemented with 2 mM NaNO_3_ and 10 mM NaHCO_3_, under white fluorescent light (30 μE m^−2^s^−1^) with a 14:10 h light:dark photoperiod, at 27 ± 1 °C, with orbital shaking. For the experiments, cultures at the exponential phase (OD_750 nm_ = 0.6–0.7, approximately 10^6^–10^7^ cellsmL^−1^) were used. The chosen cell concentration resembled that found in a bloom in a period of high cyanobacterial load, in accordance with the WHO guidance for a high probability of adverse health effects [5]. The coagulation/flocculation process was evaluated at three pHs (6, 8, and 10), adjusting the pH of the culture medium with HCl or NaOH, as appropriate.

### 2.2. Coagulation/Flocculation Materials

The chitosan was assayed at different molecular weights: low molecular weight chitosan (LMW, 250 kD avg., deacetylation degree > 90%) GP8523 Glentham Life Science, medium molecular weight chitosan (MMW, 1250 kD avg., deacetylation degree > 90%) GP8956 Glentham Life Science, and high molecular weight chitosan (HMW, >2000 kD avg.), deacetylation degree > 86%. The HMW was obtained by GIHON (kindly supplied by Dr Alberto Chevalier, GIHON, Laboratorios Químicos SRL, Mar del Plata, Argentina) from shrimp shell of local origin (Argentine Sea). Stock chitosan solutions of 0.5 % (*w*/*v*) were prepared by solubilizing the flakes in 1.5% (*v*/*v*) acetic acid at 20 °C under stirring until all the chitosan was dissolved. Bentonite clays used were type R53-1 and Bent025CS1H (pillared clay R53-1 with chitosan (HMW), prepared by the supporting of chitosan HMW on pillared bentonite) at concentrations between 0 to 30 mg L^−1^, kindly supplied by Vera Álvarez, Instituto de Investigaciones en Ciencia y Tecnología de Materiales (CONICET-Universidad Nacional de Mar del Plata, Argentina).

### 2.3. Synthesis and Characterization of N-Trimethyl Chitosan

A highly N-substituted trimethyl chitosan (TMC) was synthesized from HMW chitosan following the procedure previously described [34]. The obtained compound was analyzed by 2D NMR. The degree of substitution of TMC prepared using methyl iodide was determined by ^1^H NMR [20] at the Magnetic Resonance Laboratory, INTEQUI-CONICET, San Luis, Argentina, using a Bruker AVANCE 400 resonator (Bruker Biospin GmbH, Karlsruhe, Germany). It was operated at 400.13 MHz and 298 K. The concentration of the samples was 0.1 mg mL^−1^ in D_2_O for TMC and D_2_O/DCl for HMW chitosan.

### 2.4. C/F of M. aeruginosa Cells

Assays were conducted in test tubes and in a ‘Jar-Test’ apparatus. For this, different volumes of materials (bentonite clays, chitosan, and TMC) were added to *M. aeruginosa* cell suspensions.

#### 2.4.1. Test Tube Assays

The experiments were conducted in glass tubes (16 × 150 mm) containing 4 mL of *M. aeruginosa* suspensions that were added with different concentrations of each chitosan (0–10 mg L^−1^), TMC (0–15 mg L^−1^) or bentonite (0–30 mg L^−1^), under different pH conditions (6, 8 and 10). Controls without coagulant addition were performed in parallel. Chitosan solutions were freshly prepared for each experiment to avoid degradation [35]. After the addition of each compound, the tubes were mixed with a Vortex for 15 s and kept under stagnant conditions for 3 h. At the end of the treatment, 0.5 mL samples were taken from the top of the tubes to measure the cell density(cells/mL^−1^), the residual turbidity of the clarified zone (%RT), and the Zeta potential (ZP). In addition, cells morphology was also analyzed by SEM and TEM microscopic, and viability.

#### 2.4.2. Jar-Test Assays

C/F tests were performed using a six place multi-speed mixing unit ‘Jar-Test’ apparatus (Numak, model JT-6) with 2-L-jars. One liter of a *M. aeruginosa* culture, adjusted to pH 6 or 10, was transferred into 2-L-beakers and treated with 5 mg L^−1^ of HMW chitosan or 10 mg L^−1^ of TMC. Operating parameters were as follows: a rapid mixing at 300 rpm during 1 min, followed by 15 min at 60 rpm and 90 min of sedimentation. Then, a 1-mL-aliquot was taken from the top for counting buoyant cells.

#### 2.4.3. Determination of Cell Density, Turbidity, and Zeta Potential

After C/F treatment, the number of cells in suspension was quantified in a Neubauer counting chamber under light microscope. The percentage of residual turbidity (%RT) of the clarified culture was determined by measuring the optical density at 750 nm (OD_750_) in a UV–visible 1240 Spectrometer (Shimadzu Corporation, Kyoto, Japan). %RT was obtained by Equation (1).

(1) $$\%\mathrm{RT}=\frac{{\left({\mathrm{OD}}_{750}\right)}_{\mathrm{clarified}}}{{\left({\mathrm{OD}}_{750}\right)}_{\mathrm{control}}}\cdot 100$$

where (OD_750_)_clarified_ corresponds to the OD of the culture after treatment, and (OD_750_)_control_ was measured in the clarified culture before the addition of HMW chitosan or TMC.

The Zeta potential (ZP) was determined using a HORIBA SZ-100 Nanoparticle Analyzer. Measurements were made in quintuplicate in 6-mm electrode cells for 60 s, with a 5-s interval between measurements.

### 2.5. M. aeruginosa Cell Integrity after TMC Treatment

The viability and cell integrity were evaluated by fluorescent staining and electron microscopy, respectively.

#### 2.5.1. Viability Determination

Aliquots of 10 mL of *M. aeruginosa* cultures were treated with 10 mg L^−1^ of TMC. After 3 h, the effect on membrane integrity was evaluated in the pellets by staining with 1 µM SYTOX^®^ Green (Thermo Fisher Scientific, Waltham, MA, USA) for 10 min in the dark. The stain penetrates cells with compromised plasma membranes and binding to nucleic acids, but does not cross the membranes of live cells, thus making it an indicator of dead cells. Samples were examined under a fluorescence microscope (Nikon E600) equipped with a B-2A cube using the filter long pass for fluorescein (450–490 for excitation and 500–515 nm for emission). Cells were counted in a Neubauer chamber. Since a damaged membrane allows the stain to infiltrate, dead cells result in a green fluorescence color when analyzed under the fluorescence microscope. Negative control (untreated) and positive control (cells subjected to 70 °C for 10 min) were included. The images were captured by an Olympus DP72 digital camera, using the cellSens Entry–Microscopy Imaging Software. At least 10 random fields were taken for viability calculations in each experiment and quantified in Image [36].

#### 2.5.2. Electron Microscopy of *M. aeruginosa* Cells

A drop of 25% glutaraldehyde was added to 1.5 mL of the *M. aeruginosa* culture in an Eppendorf tube. After 20 min, the suspension was centrifuged for 5 min at 3000 rpm and the supernatant was discarded. A solution composed of 1 mL of 25% glutaraldehyde and 6 mL of saline medium was added to the pellet. After 1 h, it was centrifuged for 5 min at 3000 rpm and the supernatant was discarded. Two drops of 1.2% agarose at 45 °C were added to the precipitate and mixed with a spatula. It was allowed to harden for 20 min at 4 °C. The Eppendorf tube was cut and the sample was then transferred to a bottle with a solution consisting of 1 part saline medium and 3 parts 0.1 M sodium phosphate buffer at pH 7.4 and washed 3 times for 10 min with this solution. A 1/1 solution of osmium/sodium phosphate buffer (2% OsO_4_) was added, and the sample was left overnight. The solution was then discarded, the samples were quickly washed three times with distilled water, and a 1/1 solution of sodium phosphate buffer/uranyl acetate was added, after which the samples were incubated for 30 min in the dark. After graded ethanol (70%, 90%, and 100%) and acetone, samples were embedded in Spurr resin for 24 h at 60 °C. Thin sections were made with an ultramicrotome, which were mounted on copper grids and contrasted with uranyl acetate and lead citrate [37]. The precipitates were washed in a phosphate buffer solution and preserved for electron microscopy analysis. Morphological changes in cells exposed to HMW chitosan or TMC were assessed by Transmission Electron Microscopy (TEM) (Zeiss Libra 120, Oberkochen, Germany), and Scanning Electron Microscopy (SEM) (Zeiss Supra 55VP) in the Centro Integral de Microscopía Electrónica (CIME) UNT-CONICET, San Miguel de Tucumán, Argentina.

### 2.6. Mathematical Model

A second order quadratic model (Equation (2)) was proposed for each of the dependent variables (log N/N_0_, % RT, and ZP, determined 3 h after the addition of HMW chitosan or TMC), where Y is the predicted response, x and y are the independent variables (pH and concentrations of chitosan (mg L^−1^), respectively, and y_0_, a, b, c, and d are the coefficients of the model.

(2) $$Y=y_{0}+\mathrm{ax}+\mathrm{by}+{\mathrm{cx}}^{2}+{\mathrm{dy}}^{2}$$

The coefficient values were obtained using SYSTAT 12.0 (Systat Software, Evanston, IL, USA), with a stepwise elimination methodology to determine the significant terms of Equation (2). After fitting data with the model, the goodness of fit was evaluated considering the distribution of the residuals analysis, determination coefficient (R^2^), and the root-mean-square error (RMSE) defined in Equation (3).

(3) $$\mathrm{RMSE}=\sqrt{\frac{{\left(\mathrm{experimental}-\mathrm{predicted}\right)}^{2}}{c-d}}$$

where ‘experimental’ is the experimental data, ‘predicted’ is the value predicted by the model, c is the number of experimental data, and d is the number of parameters of the assessed model. Lower RMSE values indicate a better fit of the model to describe the data [38].

### 2.7. Statistical Analysis

All experiments were performed in triplicate and the data were expressed as the respective mean ± standard deviation of three independent determinations (SD).

Analysis of variance (ANOVA) and comparison tests according to the Fisher significant differences table (least significant difference) were applied with significance levels of 0.05. The statistical requirements for the ANOVA (normal distribution, homogeneity of variance and independence) were performed prior to executing ANOVA. The statistical software Systat (Systat version 12.0, Inc., Herndon, WV, USA) was used and the statistical significance bands were defined as 0.01 < *p* < 0.05 (*) and *p* < 0.01 (**).

## 3. Results

### 3.1. C/F Capacity of Chitosan with Different MW

**Figure 1:**
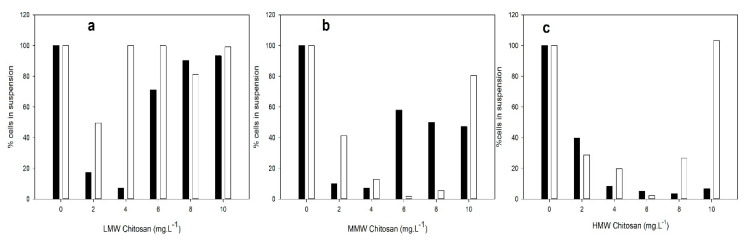
Effect of chitosan of different MW on the coagulation/flocculation of *M. aeruginosa* at the pH of the culture medium. CAAT 2005-3 cultures ■ and PCC 7806 cells cultures □ (10^7^ cells mL^−1^) were added with different amounts of chitosan: low (LMW) (**a**), medium (MMW) (**b**), and high (HMW) (**c**). After 3 h, cells were counted from an aliquot of the supernatant.

We first investigated the effect of chitosan of different molecular weights at concentrations between 2 and 10 mg L^−^^1^ on the capacity to flocculate cells from cultures of two *M. aeruginosa* strains (PCC 7806 and CAAT 2005-3) at the pH of the culture medium. As shown in Figure 1, chitosan concentrations of between 4 and 6 mg L^−^^1^ were found to be the most efficient in removing more than 90% of the buoyant cells for both strains, similar to the efficiency reported previously [11,18,23,24,39]. The ANOVA analyses showed statistically significant differences between strains (*p* = 0.0001), different types (*p* = 0.000), and concentrations of chitosan (*p* = 0.000). In addition, the pairwise comparison showed statistically significant differences between LMW chitosan and MMW chitosan (*p* = 0.000), LMW chitosan and HMW chitosan (*p* = 0.000), and MMW chitosan and HMW chitosan (*p* = 0.000).

The response of the two strains differed according to the type of chitosan and the doses used. While HMW chitosan showed a better flocculant capacity for the native strain, both MMW and HMW chitosan were effective in removing cells from PCC 7806 cultures. In addition, we tested chitosan–bentonite prepared with a similar mass ratio used in a range between 2 and 30 mg L^−^^1^ [40]. The ANOVA analyses showed significant statistical differences between the types of chitosan–bentonite (*p* = 0.698), and different concentrations (*p* = 0.666). The concentrations assayed were not effective for removing cyanobacterial cells (Figure S1, Supplementary Material).

As HMW chitosan showed a good performance, it was chosen to scale in a Jar-Test assay, using vessels with one liter of culture of *M. aeruginosa* strains PCC 7806 or CAAT 2005-3. Chitosan at a concentration of 5 mg L^−^^1^ was added, and the removal effectiveness was greater than 95% (Figure S2, Supplementary Material).

### 3.2. Characterization of Trimethyl Chitosan (TMC)

**Figure 2:**
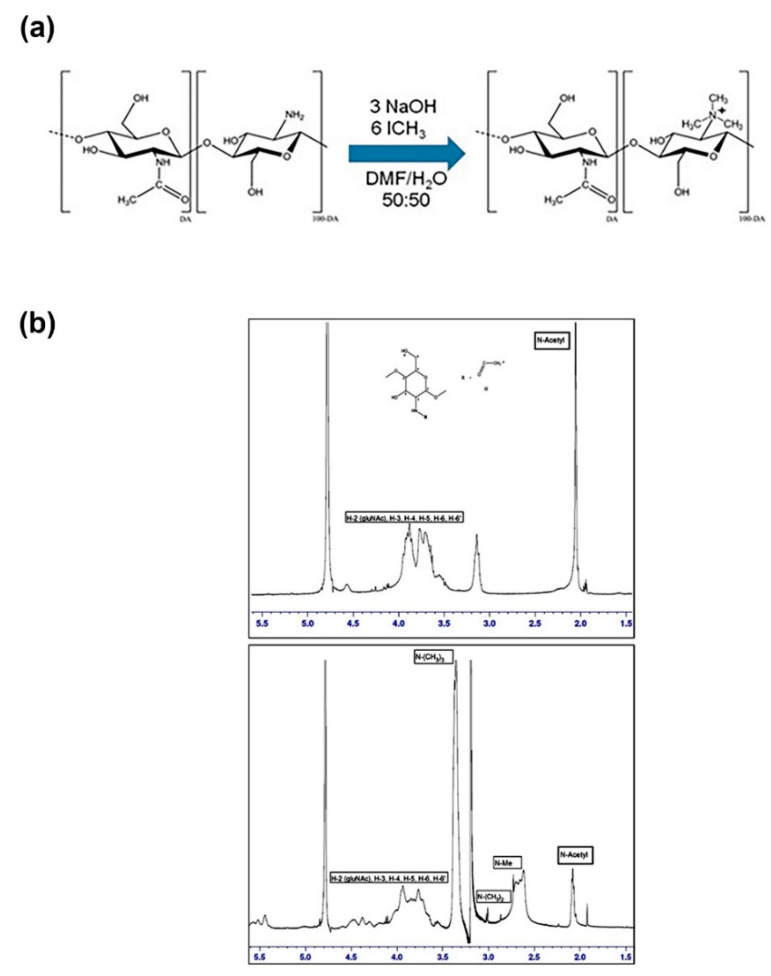
Trimethyl chitosan characterization. (**a**) Scheme of the synthesis and TMC structure. (**b**) ^1^H NMR spectrum (600 MHz) of chitosan (upper panel) and TMC in D_2_O at 65 °C. Acetone was used as a reference (2.2 ppm). The main differential groups are indicated on the spectrum.

TMC, a well-studied N-quaternary water-soluble chitosan derivative, was obtained from the HMW chitosan to give a stable positive charge polymer [34], allowing its use independently of pH. The structural changes in the chitosan molecules were confirmed by FT-IR spectroscopy and characterized by 2D NMR (Figure 2 and Figure S3, Supplementary material).

The strong signal at c.a. 3.3 ppm in TMC corresponding to N-methyl groups is not observed in HMW chitosan, which reflects a high degree of N-methylation, a condition produced by the TMC [34,41,42].

### 3.3. Determination of the C/F Capacity of TMC in Jar-Test Assays

**Figure 3:**
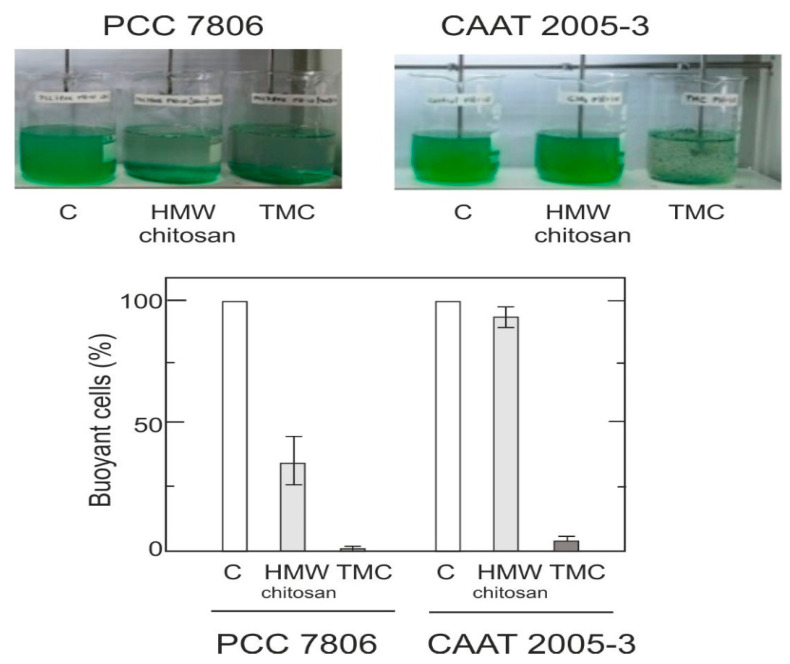
Removal of *M. aeruginosa* cells by HMW chitosan and TMC in a Jar-Test experiment. The capacity of 5 mg L^−1^ HMW chitosan at pH 10 to remove cells of two *M. aeruginosa* strains (PCC 7806 and CAAT 2005-3) was assayed in a Jar-Test experiment.

The cell removal capacity of TMC on two *M. aeruginosa* strains (PCC 7806 and CAAT 2005-3) was assayed at pH 10 (a pH close to the values present in blooms [43,44,45]), in Jar-Test vessels (Figure 3). The percentage of buoyant cells with respect to the control was calculated after each treatment. The ANOVA analyses showed significant statistical differences between strains (*p* = 0.0000) and different types of chitosan (*p* = 0.000). In addition, there were significant statistical differences between HMW chitosan and TMC (*p* = 0.000).

### 3.4. Removal Capacity of M. aeruginosa by HMW Chitosan and TMC

**Figure 4:**
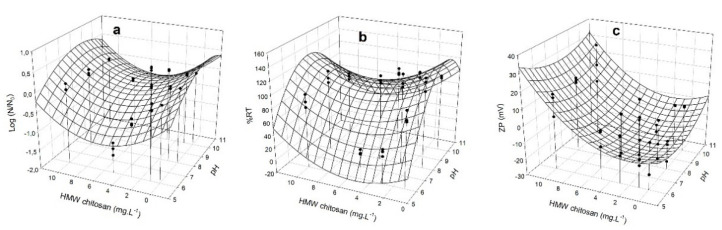
Response surface plot for *M. aeruginosa* PCC 7806 of: (**a**) log (N/N_0_), where N and N_0_ are the number of cells/mL^−1^after and before the treatment, respectively; (**b**) %RT removal; and (**c**) ZP of the clarified zone, as a function of different concentrations of HMW chitosan (mg L^−1^) and pHs. (•) experimental measurements.

**Figure 5:**
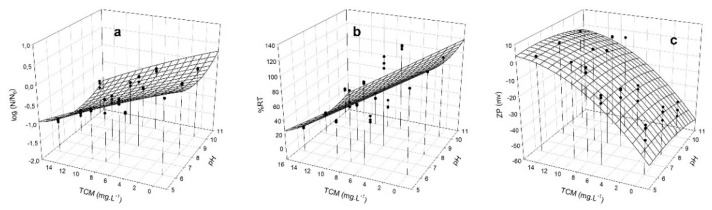
Response surface plot for *M. aeruginosa* PCC 7806 of: (**a**) log (N/N_0_); (**b**) %RT removal; and (**c**) ZP of the clarified zone, as a function of different concentrations of TMC (mg L^−1^) and pHs. (•) experimental measurements.

**Figure 6:**
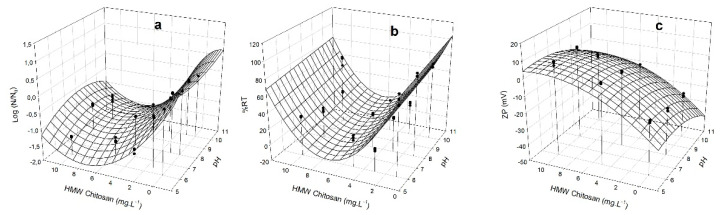
Response surface plot for *M. aeruginosa* CAAT 2005-3 of: (**a**) log (N/N_0_), (**b**) %RT removal; and (**c**) ZP of the clarified zone, as a function of different concentrations of HMW chitosan (mg L^−1^) and pHs. (•) experimental measurements.

**Figure 7:**
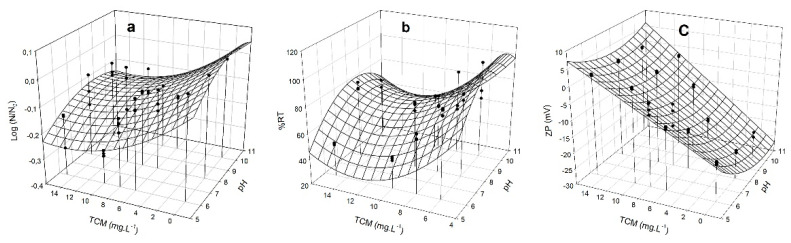
Response surface plot for *M. aeruginosa* CAAT 2005-3 of: (**a**) log (N/N_0_); (**b**) %RT removal; and (**c**) ZP of the clarified zone, as a function of different concentrations of TMC (mg L^−1^) and pHs. (•) experimental measurements.

Since the flocculation process depends on the surface charge of the cells, we measured the Zeta potential (ZP) [24] together with changes of log (N/N_0_), and %RT in the supernatants at different doses of HMW chitosan and TMC, at different pHs. ZP gives an idea of charge neutralization and therefore of flocculation, since high ZP values reflect significant electrostatic repulsions between particles and small sedimentation volumes [46]. The obtained data enabled us to generate a model to predict the best condition of coagulation/flocculation. The dependence of the different responses (log (N/N_0_), %RT, and ZP) on HMW chitosan and TMC concentrations was analyzed in *M. aeruginosa* PCC 7806 (Figure 4 and Figure 5) and in CAAT 2005-3 (Figure 6 and Figure 7). The experimental measurements overlapped in Figure 4, Figure 5, Figure 6 and Figure 7 show the satisfactory agreement of the experimental values with the predicted functions describing the response surface plot of each dependent variable (log (N/N_0_), %RT, and ZP).

The coefficients (y_0_, a, b, c, and d) were obtained by applying the model of Equation (2) and the stepwise elimination methodology with the SYSTAT 12.0 software. The coefficient of determination (R^2^) and the root-mean-square error (RMSE) applied to the different responses showed a good fit of the model to the experimental data (Table 1).

The parameters y_0_, a, b, c, and d allow for prediction of the values of the dependent variables (log (N/N_0_), %RT, and ZP) using Equation (2), at any independent variable (pH and chitosan concentration values). The surface responses show that there is an optimal minimum value in the range of the study’s independent variables.

The optimal minimum values for ZP should be fixed close to 0 mV, allowing a greater chance of aggregation.

For *M. aeruginosa* CAAT 2005-3, the minimum values of log(N/N_0_), %RT, and ZP (−1.5, 0.79%, and 0.79 mV, respectively) were reached at concentrations of HMW chitosan ranging between 6.7–7.7 mg L^−^^1^ and pH ranging between 6.1–7.2 mg L^−^^1^. For *M. aeruginosa* PCC 7806, the minimum values of log (N/N_0_), %RT, and ZP (−0.54, 49%, and 0.122 mV, respectively) were reached at concentrations of HMW chitosan between 5.1 and 5.9 mg L^−^^1^ and pH = 6.1 mg L^−^^1^. The optimum pH range with HMW chitosan was 6.1 to 7.2.

For *M. aeruginosa* CAAT 2005-3, the minimum values of log (N/N_0_), %RT, and ZP (−0.18, 50%, and 0.25 mV, respectively) were reached at concentrations of TMC ranging between 10 to 14 mg L^−^^1^ and pH in the range of 6.1 to 8.7. For *M. aeruginosa* PCC 7806, the minimum values of log (N/N_0_), %RT, and ZP (−1.27, 10%, and 0.17 mV, respectively) were reached at concentrations of TMC between 11 and 14 mg L^−^^1^, and pH = 8.0 to 9.8 mg L^−^^1^.

### 3.5. Effect of TMC on M. aeruginosa Cell Membrane Integrity

Since the C/F process may exert physiological or chemical stress on cyanobacterial cells [47], we investigated whether TMC presence could cause damage to membrane integrity and consequent release of cell content. The effect of TMC on *M. aeruginosa* cell membrane was evaluated first by staining with SYTOX^®^ Green nucleic acid stain (Molecular Probes, Waltham, MA, USA) and then by electron microscopy.

**Figure 8:**
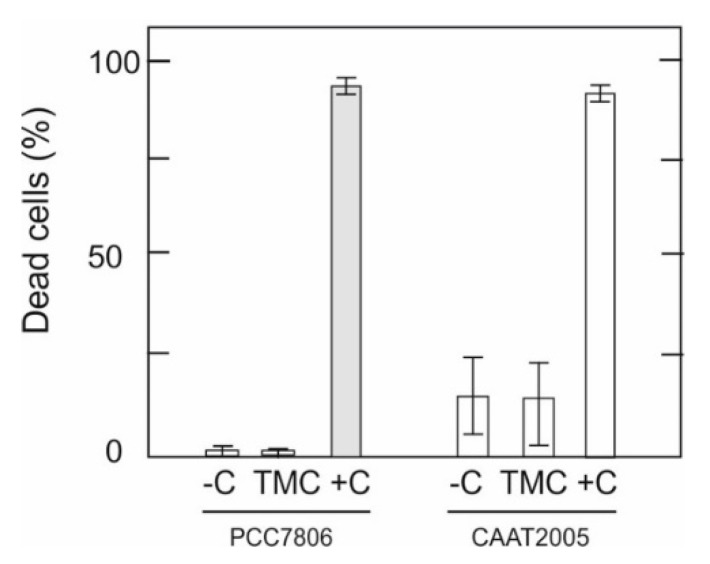
*M. aeruginosa* viability after TMC treatment. Cell death in two *M. aeruginosa* strains (PCC 7806 and CAAT2005-3) was evaluated in cell pellets after TMC treatment. Cells were stained with SYTOX Green, examined, and counted under light and fluorescence microscopy. SYTOX-positive cells were interpreted as being dead cells. Negative control (−C, untreated cells) and positive control (+C, cells subjected to 70 °C for 10 min) were included.

The percentage of viable (SYTOX negative control) to nonviable (SYTOX positive control) cells was determined after the flocculation treatment. As shown in Figure 8, TMC treatment exhibited a similar result to non-treated cells (negative control), indicating that the flocculant could be used without cell damage.

**Figure 9:**
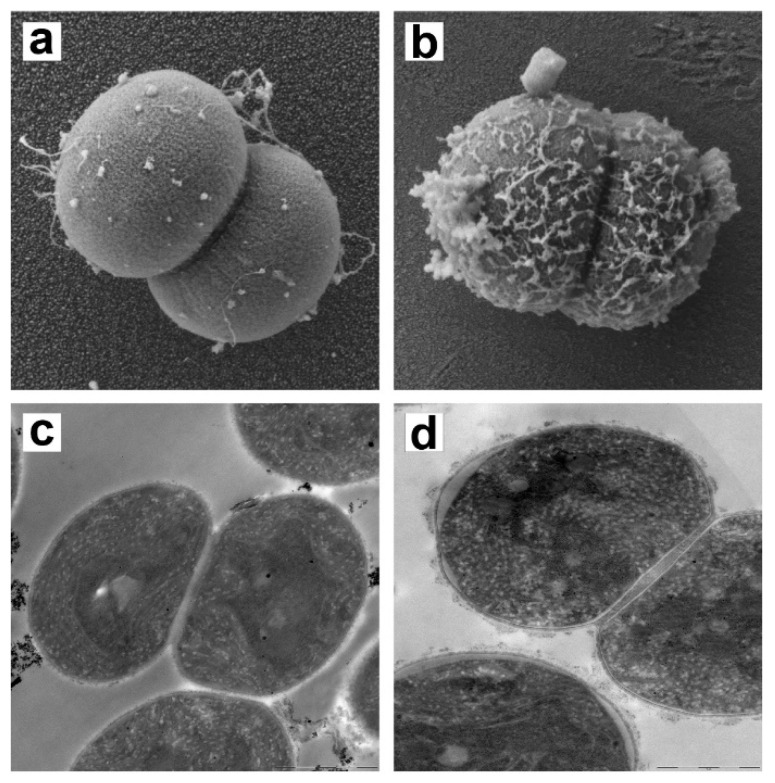
SEM and TEM of *M. aeruginosa* after C/F treatment. (**a**) SEM *M. aeruginosa* sp. PCC 7806 control, Mag: 50,000 K; (**b**) SEM *M. aeruginosa* treated with TMC, Mag: 50,000 K; (**c**) TEM *M. aeruginosa* sp. PCC 7806 control; (**d**) TEM *M. aeruginosa* treated with TMC.

In addition, SEM and TEM micrographs were taken to analyze *M. aeruginosa* cell changes after TMC treatment (Figure 9). Compared to the non-treated control, the membrane of *M. aeruginosa* cells remained intact, and morphology did not change after C/F; that is, the TMC treatment did not cause damage to the cyanobacterial cells.

## 4. Discussion

As a contribution to the elimination of cyanobacterial cells present in fresh water sources intended for human consumption, we explored the use of different chitosan products, which are biodegradable and environmentally friendly coagulants/flocculants. The chitosan assayed (LMW, MMW, and HMW) showed different cell removal capacity depending on the molecular weight, concentration, and *M. aeruginosa* strain used. The greatest effectiveness in removing cyanobacterial cells was achieved with HMW chitosan, both in test tubes and in the Jar-Test. In test tube experiments, the most effective chitosan concentration was around 4 to 6 mg L^−^^1^, which allowed for the removal of 90% of suspended cells (Figure 1). A volume of 5 mg L^−^^1^ HMW chitosan removed 95% of cells in Jar-Test assays (Figure 3 and Figure S2). However, this finding cannot be generalized and the appropriate concentration for C/F should be evaluated in each case, based on the predominant *Microcystis* sp. strain in the bloom. The chitosan–bentonite concentrations tested were not effective in removing cells in the range of 0–30 mg L^−^^1^, which contrasts with previously reported results [40]. This discrepancy could be attributed to differences in the type of chitosan used to prepare the chitosan–bentonite composite.

Considering that the solubility of chitosan depends on the pH, it is effective at a pH lower than 7, and that blooms have usually alkaline pHs, it was decided to synthesize TMC from HMW chitosan extracted from residues of the fishing industry. We were able to confirm that the introduction of a permanent positively charged group to chitosan vastly increases its water solubility. This is in line with previous reports that found TMC to be soluble up to 10 g L^−^^1^ over a pH range of 1–13 [48,49,50]. From the TMC and the HMW chitosan used in its synthesis, we generated a data set evaluating the removal of buoyant cells of two *M. aeruginosa* strains after varying the pH and flocculant concentration. A second order quadratic model was used to compare the effectiveness of cell removal, with the statistical parameters R^2^ and RMSE (Table 1) showing a good fit. 

The response surface plots show that that there is an optimal pH and a minimum concentration value in the range of the studied independent variables. Optimal doses of HMW chitosan and TMC, as well as optimal pH ranges, were obtained by applying a second order quadratic model to reach minimum values of %RT, log (N/N_0_), and ZP. Since in the case of TMC the optimal pH range for C/F is between 6.1 and 9.8, we conclude that it could be suitable for treating water contaminated with blooms, ruling out the use of HMW chitosan, which is effective only in pH ranges between 6.1 and 7.2 (Figure 4, Figure 5, Figure 6 and Figure 7). Although this study was carried out with cells in culture medium, our results indicate that TMC in an optimal concentration range between 10 and 14 mg L^−^^1^ could be used to remove *M. aeruginosa* cells not only from water supply treatment plants, but also from blooms that have generally alkaline pHs [43,44,45]. The different C/F behavior obtained with each strain and the two chitosans assayed could be ascribed to the different morphological characteristics of the cells of strains PCC 7806 and CAAT 2005-3.

A significant finding of the present paper upon evaluation of the viability and cell membrane integrity of *Microcystis* cells after treatment with TMC (Figure 8) is that the C/F process does not cause cell damage. This agrees with the electron microscopy images, the morphology characterized by TEM being in line with that obtained by SEM in *M. aeruginosa* treated with TMC (Figure 9). The fact that the *M. aeruginosa* cells remained intact in the coagulated system augurs well for the use of TMC as a C/F agent, as it indicates that undesirable harmful cellular components would not be released into the medium under these circumstances. However, this promising outlook requires further research to determine whether cell lysis might occur during floc storage, with the consequent release of toxins. It is therefore necessary to determine the microcystin concentration in the water after TMC treatment, the factors that influence cell lysis, and the mechanisms involved.

## 5. Conclusions

High *M. aeruginosa* cell removal was obtained in test tube assays by C/F with TMC in the range of 10–14 mg L^−1^, at a pH range found in blooms.

TMC was effective in removing the cells in suspension (˃95%) in both strains at pH 10 in a Jar-Test assay.

TMC did not cause cell damage under the conditions tested, which would indicate no leakage of internal metabolites within 3 h of starting treatment.

## Figures and Tables

**Table 1:** Coefficients determined by regressions using response surface methodology (RMSE) for each analyzed response and statistical parameters (determination coefficient (R^2^).

Strain	Flocculant		Coefficients						
			y_0_	a	b	c	d	R^2^	RMSE
PCC 7806	HMW chitosan	Log (N/N_0_)	−3.84 ± 1.33	0.911 ± 0.3444	−0.165 ± 0.041	−0.051 ± 0.002	0.0162 ± 0.003	0.487	0.271
		% RT	−133.8 ± 10.11	−9.08 ± 0.25	−33.73 ± 6.10	56.23 ± 1.67	2.28 ± 0.37	0.775	0.426
		ZP	37.19 ± 44.33	−14.90 11.50	2.34 ± 1.40	1.02 ± 0.72	0.06 ± 0.13	0.755	7.281
	TMC	Log (N/N_0_)	1.36 ± 0.14	−0.08 ± 0.03	−0.01 ± 0.001	0.005 ± 0.003	−1.10^−4^ ± 2.10^−5^	0.791	0.030
		% RT	209.78 ± 114.8	−24.57 ± 29.64	−6.15 ± 2.09	1.39 ± 1.84	0.002 ± 0.001	0.656	0.615
		ZP	−50.23 ± 38.79	4.50 ± 10.01	4.71 ± 0.70	−0.35 ± 0.62	−0.13 ± 0.04	0.773	7.892
CAAT 2005-3	HMW chitosan	Log (N/N_0_)	−3.18 ± 0.80	0.79 ± 0.20	−0.39 ± 0.02	−4.75 ± 0.01	0.02 ± 0.002	0.912	0.164
		% RT	79.20 ± 8.14	2.14 ± 0.22	−28.71 ± 3.57	0.008 ± 0.001	2.235 ± 0.314	0.711	14.842
		ZP	−42.60 ± 16.51	8.32 ± 4.27	4.27 ± 0.48	−0.66 ± 0.27	−0.204 ± 0.047	0.955	2.490
	TMC	Log (N/N_0_)	−0.50 ± 0.21	0.11 ± 0.08	−0.02 ± 0.001	−0.006 ± 0.00	8.9.10^−4^ ± 3.10^−5^	0.535	0.063
		% RT	−20.34 ± 6.24	42.49 ± 15.12	−16.67 ± 4.41	−2.48 ± 0.94	0.70 ± 0.20	0.544	10.660
		ZP	14.01 ± 18.30	−8.25 ± 4.72	1.60 ± 0.33	0.45 ± 0.29	−1.10^−3^ ± 1.10^−4^	0.837	3.721

## Data Availability

Not applicable.

## Supplementary Materials

The following supporting information can be downloaded at: https://www.mdpi.com/article/10.3390/microorganisms10102052/s1, Figure S1: Effect of different clays on the coagulation/flocculation of *M. aeruginosa* CAAT-2005-3 cells. Cya-nobacterial cultures (10^7^ cells mL^−1^) were added with different amounts of clay Bent R53-1 and Bent 025CS1H). After 3 h, cells were counted from an aliquot of the supernatant. 100% corre-sponds to the number of cells before addition of the polymer. Figure S2: Removal of *M. aeruginosa* cells by HMW chitosan in a Jar test experiment. The capacity of 5 mg L^−1^ HMW chitosan at pH 6 to remove cells of two *M. aeruginosa* strains (PCC 7806 and CAAT 2005-3) was assayed in a Jar-test experiment. Figure S3: COSY-NMR spectra for HMW-chitosan (a) and TMC obtained after 48 h of hydrolysis (b).
