# Supplementary material for: A Promising Use of Trimethyl Chitosan for Removing Microcystis aeruginosa in Water Treatment Processes
Source: Microorganisms. 2022 Oct 18;10(10):2052. doi: 10.3390/microorganisms10102052 (PMC9610100; doi:10.3390/microorganisms10102052)
Supplement: Supplementary file 1 [file microorganisms-10-02052-s001.zip › microorganisms-1917952-supplementary.pdf]

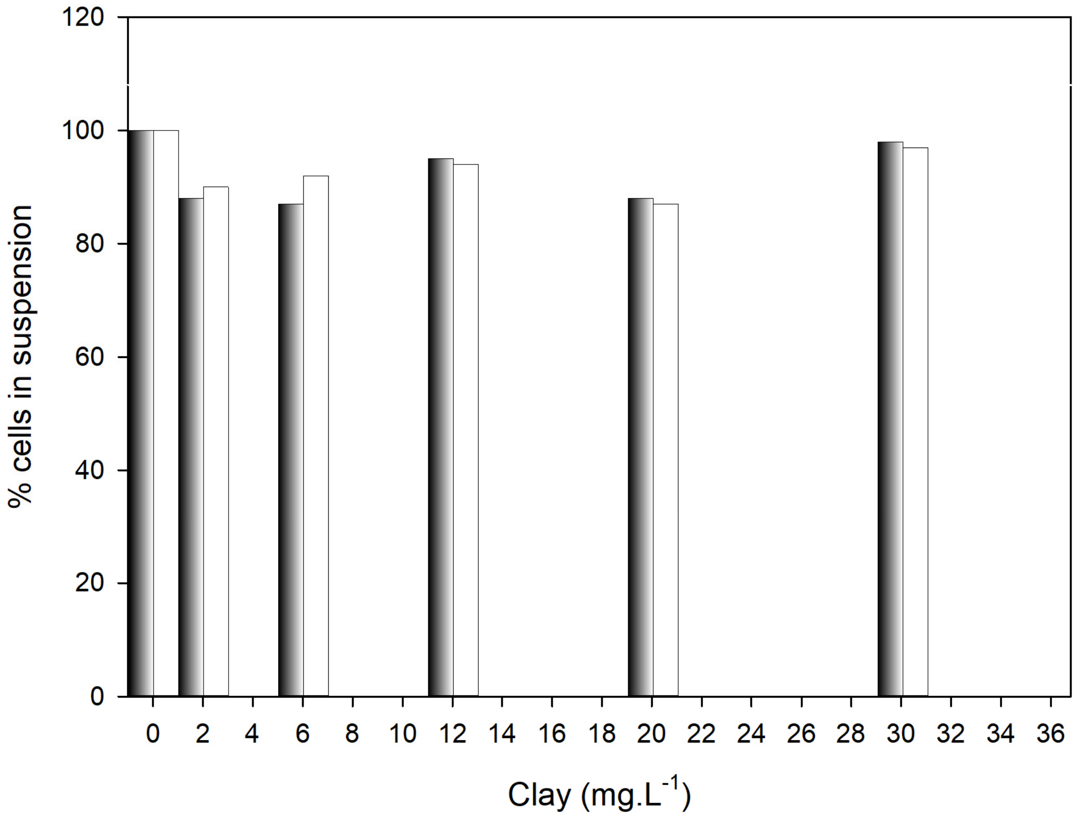

Figure 1. Effect of different clays on the coagulation/flocculation of M. aeruginosa CAAT-2005-3 cells. Cya-nobacterial cultures (107 cells mL^{-1}) were added with different amounts of clay Bent R53-1 and Bent 025CS1H). After 3 h, cells.were counted from an aliquot of the supernatant. 100% corre-sponds to the number of cells before addition of the polymer.

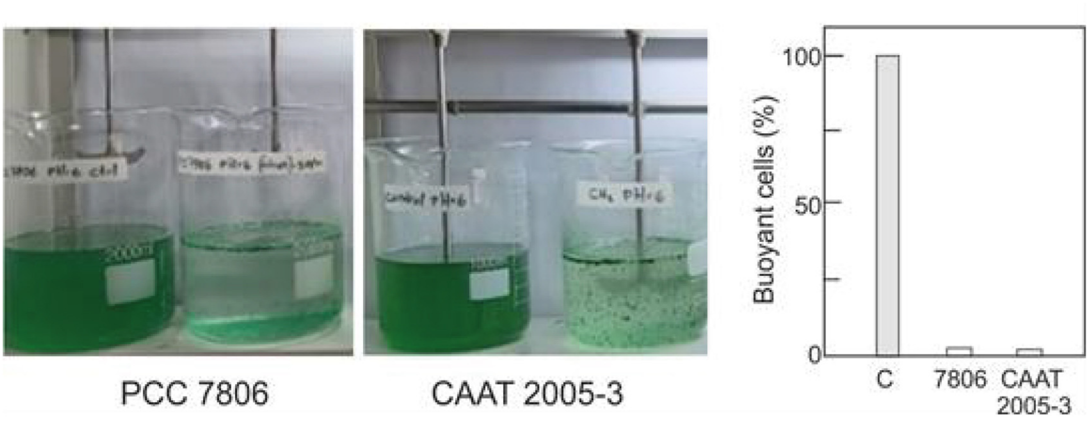

Figure S2: Removal of M. aeruginosa cells by HMW chitosan in a Jar test experiment. The capacity of 5 mg L^{-1} HMW chitosan at pH 6 to remove cells of two M. aeruginosa strains (PCC 7806 and CAAT 2005-3) was assayed in a Jar-test experiment.

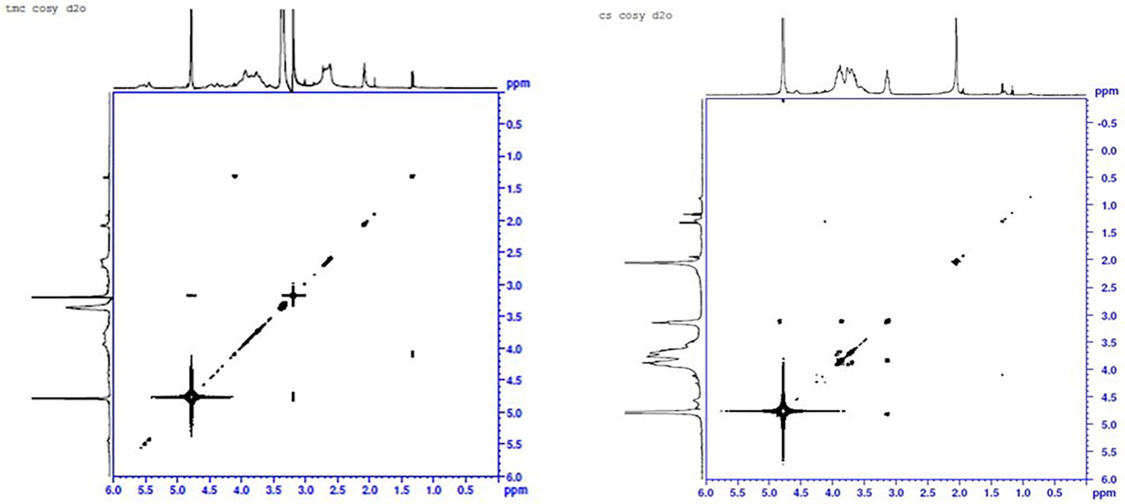

(**a**) (**b**)

Figure S3: COSY-NMR spectra for HMW-chitosan (a) and TMC obtained after 48 h of hydrolysis (b).
